# *Epipactis bucegensis*—A Separate Autogamous Species within the *E. helleborine* Alliance

**DOI:** 10.3390/plants12091761

**Published:** 2023-04-25

**Authors:** Nora E. Anghelescu, Mihaela Balogh, Lori Balogh, Nicoleta Kigyossy, Mihaela I. Georgescu, Sorina A. Petra, Florin Toma, Adrian G. Peticilă

**Affiliations:** 1 Faculty of Horticulture, University of Agronomic Sciences and Veterinary Medicine of Bucharest, 59 Mărăști Blvd, District 1, 011464 Bucharest, Romania; 2 Association “Comori de pe Valea Prahovei”, 2 Avram Iancu St., 106100 Sinaia, Romania

**Keywords:** *Epipactis bucegensis*, comparative morphology, pollination biology, taxonomy, ultrastructure, micromorphology, orchids

## Abstract

A new species of *Epipactis* from Bucegi Natural Park ROSCI0013, Southern Carpathians, Central Romania is described. Three medium-sized populations of *Epipactis bucegensis* (65–70 individuals in total) were discovered in the south-eastern, subalpine area of the park. To properly describe and distinguish the newly found taxon from other Romanian *Epipactis*, 37 morphological characters were measured directly from living plants and flowers. Moreover, a detailed taxonomic treatment and description with corresponding colour photos and line drawings illustrations of the holotype are also included. *Epipactis bucegensis* is an obligate autogamous species that partially resembles *Epipactis muelleri,* from which it differs in the basal distribution of leaves on the stem (vs. median distribution); near-erect leaf posture (vs. horizontally spread, arched downwards); lanceolate–acuminate, yellowish-green leaves (vs. oval–elongate, vivid-green leaves); bipartite labellum lacking the mesochile (vs. tripartite labellum); crimson-red, wide, ovoid–elongated, flattened hypochile (vs. dark-brown to black roundish hypochile); triangular, white epichile with a sharply tapering apex (vs. heart-shaped, greenish-yellow epichile with obtuse, roundish apex); and two wide-apart, purple, pyramidal calli (vs. two closely placed, attenuated, mildly wrinkled, greenish-yellow calli). *Epipactis bucegensis* is easily distinguished from all other European *Epipactis* taxa by the bipartite, wide labellum that lacks the mesochile. In addition, information regarding its distribution (maps), habitat, ecology, phenology and IUCN conservation assessments are provided.

## 1. Introduction

Genus *Epipactis* Zinn, 1757 belongs to Tribe Neottieae Lindl., 1826, Subfamily Epidendroideae Lindl., 1821, Family Orchidaceae Juss., 1789. The generic name, *Epipactis*, originates in the ancient Greek word *epipaktís*, a name given for the first time by Greek philosopher and botanist Theophrastus of Eresos (ca. 371–ca. 287 B.C.E.) to a herbaceous plant that curdled milk, possibly a member of the highly poisonous, unrelated genera of invasive plants *Helleborus* (Family Ranunculaceae, known as Hellebores) and *Veratrum* (Family Melanthiaceae, known as the False Hellebores). Since *Epipactis* orchids have been associated with the poisonous, invasive Hellebores from ancient times, the generic vernacular name of this genus remains, to this day, the Helleborines [1].

Depending on the species concept of various authorities, the number of *Epipactis* species varies considerably, showing drastic fluctuations over time: Richards (1982) counts 25 species [2]; Delforge (1995, 1996) counts 36 species [3,4], a number he later increased to 56 species [5,6] and then to 65 species [7]. *Genera Orchidacearum* considered the genus to contain approximately 15 species [1], which is in line with Kuhn et al. (2019), who mention 16 species and several infraspecific taxa [8]. Nevertheless, in recent years, the taxonomy of the genus has suffered further inflation, with Griebl and Presser (2021) reporting approximately 75 species [9], a number increased to approximately 95 species in the comprehensive online monograph/database of the *Arbeitskreis Heimische Orchideen Bayern* e.V. [10].

According to the most recent classification [5] the *Epipactis* genus is divided into two monophyletic sister sections that are differentiated by the shape and structure of the labellum: 1. Section *Arthrochilium* (Irmisch) Beck 1890, characterised by a mobile (hinged) epichile and a hypochile with lateral lobes (not cup-shaped), represented by the *Epipactis palustris* (L.) Crantz and *Epipactis veratrifolia* Boiss. and Hohen. groups. 2. Section *Euepipactis* Irmisch (1842), more commonly known as the *Epipactis helleborine* alliance/aggregate, characterised by a cup-shaped hypochile lacking lateral lobes and an immobile (fixed) epichile, represented by seven groups: *E. phyllanthes* (hairless stems and a greenish-yellow pedicel base), *E. leptochila* (pubescent stem and green pedicel base)*, E. purpurata* (hairy stem and purplish plants), *E. helleborine* (hairy to villous stem and purple-pigmented pedicel base), *E. atrorubens* (hairy stems and violet-pigmented pedicels), *E. tremolsii* (intermediate between *E. atrorubens* and *E. helleborine*) and *E. albensis* (intermediate between *E. leptochila* and *E. phyllanthes*) groups. 

The *Epipactis* genus is mainly distributed in the temperate, meridional and sub-meridional zones across Europe, eastward through Asia and Japan and southward to tropical Africa [1]. The only endemic African representative is *Epipactis africana* Rendle and the only American endemic species is *Epipactis gigantea* Douglas ex Hook., known as the Giant Helleborine [11]. Section *Euepipactis* has a natural distribution that is confined to Eurasia, though the most widespread species, *E. helleborine* sensu stricto (s.s.), has become an increasingly common occupier of anthropogenic habitats [12], to the extent that it is viewed as an invasive adventive in North America [13,14,15]. 

In general, *Epipactis* orchids are robust and tall, up to 1 m in height, e.g., *Epipactis helleborine* (L.) Crantz and *Epipactis distans* Arv.-Touv., but there are also smaller representatives with their flowering stem reaching only a few centimetres in height, e.g., 5–7 cm in *Epipactis tallosii* A.Molnár and Robatsch, *Epipactis microphylla* (Ehrh.) Sw. or *Epipactis exilis* P. Delforge. The leaves are usually dark green, wide, with prominent venation and ovate–elongated in shape. The inflorescences are large, consisting of many small, variously coloured flowers [16]. The middle petal, the labellum, consists of three parts, the base—the *hypochile*—cup-shaped, nectar-secreting (in allogamous species) or nectarless (in autogamous species); the junction between the hypochile and the epichile—the *mesochile* [17], usually very narrow, hinged and well-defined; and the terminal part—the *epichile*—heart-shaped, adorned with various calli and keels on its surface [5,7]. Temperate *Epipactis* are summer-green orchids that develop their leaves at the beginning of summer (June–August, in the Northern Hemisphere) and lose them between the end of summer and the beginning of autumn (September–November) [18]. Besides the green-leaved (photosynthetic) species, our field studies included partially mycoheterotrophic species, e.g., *Epipactis purpurata* Sm., and fully mycoheterotrophic (holomycotrophic) taxa, such as the chlorotic forms (achlorophyllous) of *Epipactis helleborine* (L.) Crantz, *Epipactis microphylla* (Ehrh.) Sw. and *Epipactis purpurata* Sm. f. *rosea* (Erdner) P.Delforge [19]. Fully or partially mycoheterotrophic species arose many times in the evolution of the tribe Neottieae [20,21,22].

*Epipactis* species flower from June to August [19]. They are mostly found in very diverse habitats, from sandy beaches to open spaces, in deciduous (beechwoods) or coniferous forests, on roadsides, in meadows or even flooded habitats, on alkaline to neutral (rarely mildly acidic), moist to dry substrates. 

Collectively, the genus consists of autogamous (either facultative or obligate) and allogamous species. The autogams are nectarless and self-pollinate without the need for insect pollinators. The allogams are nectar-rewarding and depend on insects to cross-pollinate (entomophilous) [23,24,25,26,27,28,29].

**Figure 1:**
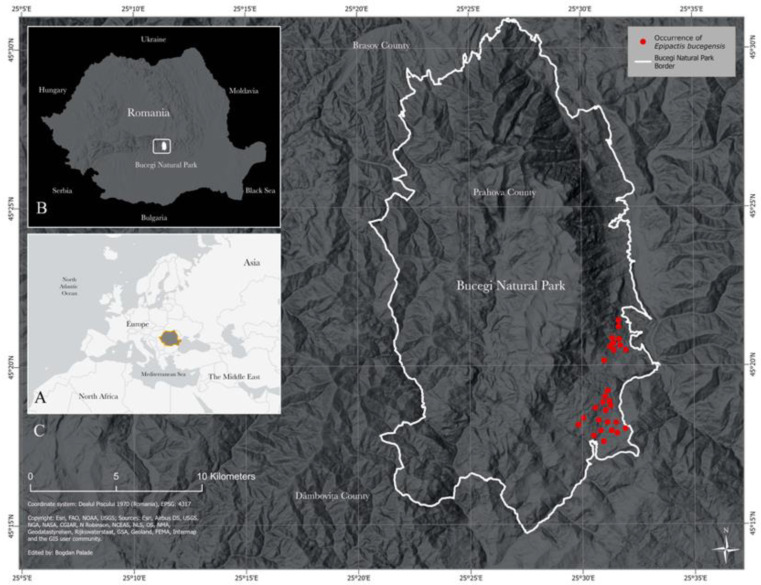
Geographical distribution of *Epipactis bucegensis* N.Anghelescu, L.Balogh and M.Balogh. (**A**) Map of greater Europe, North Africa, the Middle East and Asia; Romania (dark-grey coloured) is situated in the south-eastern part of Europe. (**B**) Map of Romania and its neighbouring countries. (**C**) Map of Bucegi Natural Park (BNP) ROSCI001, Southern Carpathians, Central Romania. Known locations of the type specimens (holotype) *E. bucegensis*, with an extent of occurrence (EOO) of 10–15 km^2^ and an area of occupancy (AOO) of ca. 45–50.5 km^2^ (red dots). Map created by Bogdan Palade.

In this paper, we describe and illustrate a new autogamous species within the *E. helleborine* alliance named *Epipactis bucegensis.* The first encounter with *Epipactis bucegensis* took place in July 2009 during an orchid field study in the south-eastern part of Bucegi Natural Park, Southern Carpathians (Figure 1C, red dots). 

At first sight, in the harsh light of the melting-hot summer days, the plants looked rather like a peculiar group of yellowish, withered *Epipactis helleborine* (L.) Crantz, perfectly camouflaged among the brownish, grassy, surrounding vegetation. However, the most striking features were the elongated inflorescences bearing several inconspicuous, creamy-white, closed flowers hanging on the pendant, yellowish ovaries, a clear indication of an autogamous species, different from the common, allogamous *Epipactis helleborine* (L.) Crantz, which is sporadically found in the area. After a closer examination, which involved manually opening several flowers, we also noticed the unusual, unique structure of the nectarless labellum, which completely lacked the middle narrowing junction, a feature that differentiated it from any other Romanian *Epipactis* species. Furthermore, the absence of the viscidium and the crumbling, disintegrating pollinia reinforced our initial supposition of a distinct, autogamous taxon. Furthermore, in the summer of 2022, several expeditions to Bucegi Natural Park were made, and during these field trips, two new populations of the same taxon were discovered. The most distinctive morphological features proved to be highly preserved and consistent, the new specimens showing little to no variation regarding the yellowish aspect of the plants, the creamy-white closed flowers and the nectarless labellum completely lacking the middle junction. Undeniably, the newly discovered populations confirmed, once more, the occurrence of a persistent, new species, well-established within the south-eastern area of the park. Consequently, we chose to formally describe this new taxon as *Epipactis bucegiana*, with the confidence that, in the years to come, more undiscovered populations will be revealed within the park’s greater area. Additionally, we provide information about its geographical distribution, habitat, ecology, phenology and IUCN conservation status, together with illustrations and photographs based on living specimens (the holotype). 

## 2. Results

### 2.1. Sites Studied 

The study sites were on wet to dry, calcareous substrates, next to deciduous to mixed woodland; altitude between 700–1100 m a.s.l. The populations occurred in sunny meadows and pasturelands, neighbouring margins of mixed forests covering subalpine slopes, close to urban sites (Figure 1C, red dots).

### 2.2. Morphological Comparisons

**Figure 2:**
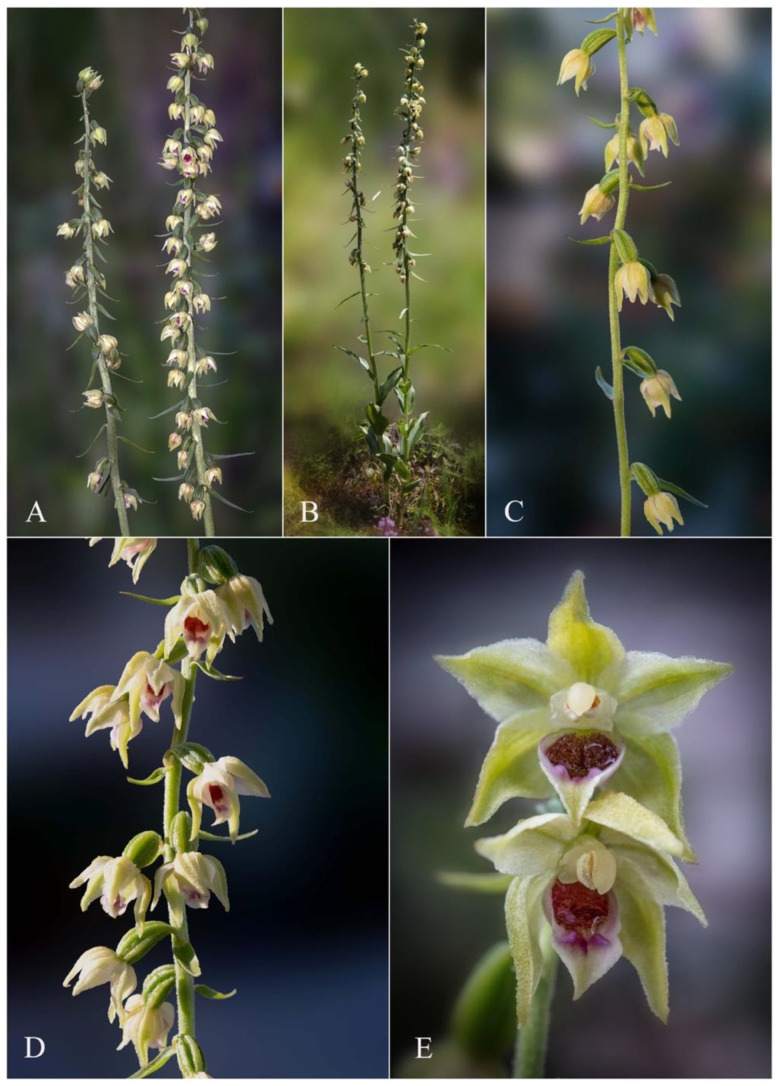
*Epipactis bucegensis* N.Anghelescu, L.Balogh and M.Balogh. (**A**) Inflorescences of sibling individuals; inflorescences are lax to dense racemes, floriferous; most of the cleistogamous flowers show swollen ovaries, a sign of early pollination in bud stages (before anthesis). (**B**) *Epipactis bucegensis* in its natural habitat. (**C**) Detail of the inflorescence at full anthesis with pendant, cleistogamous flowers (closed flowers). (**D**) Inflorescence at full anthesis with hand-opened flowers. (**E**) Topmost flowers, hand-opened, show minor traces of nectar; older flowers are completely devoid of nectar. Photographs by Lori Balogh ((**A**) 17 July 2022, BNP, Romania) and Nora E. Anghelescu ((**B**–**E**) 19 July 2022 BNP, Romania).

**Figure 3:**
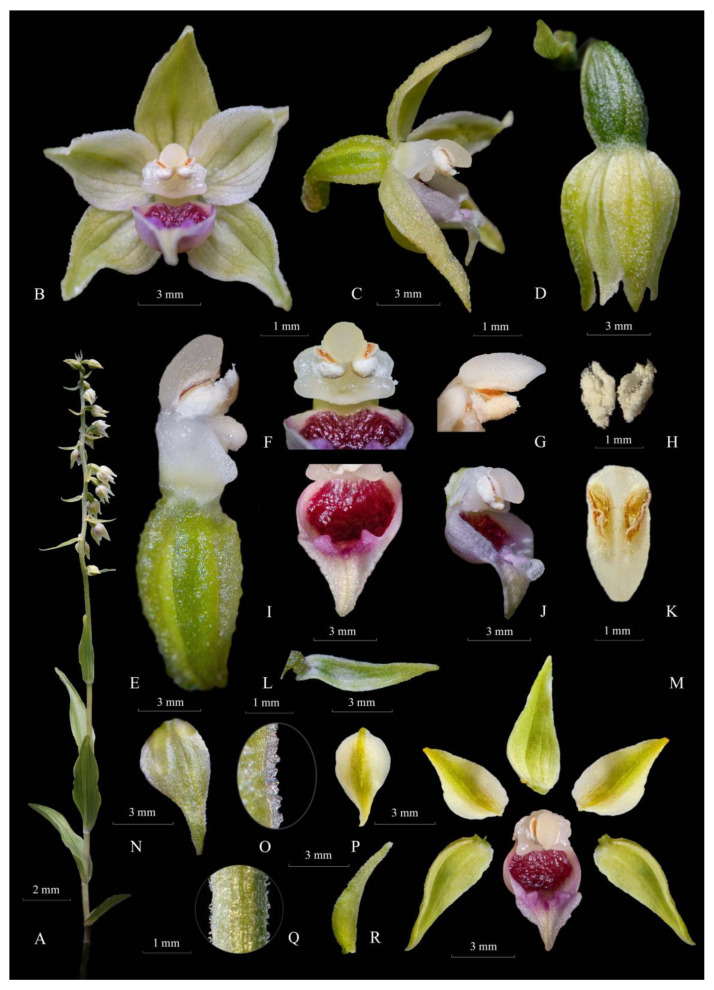
Composite dissection plate (CDP) of *Epipactis bucegensis* N.Anghelescu, L.Balogh and M.Balogh. (**A**) Habitus and leaves. (**B**) Flower—frontal view (flower hand-opened). (**C**) Flower—side view (flower hand-opened). (**D**) Cleistogamous flower at full anthesis. (**E**) Gynostemium and ovary—lateral view. (**F**) Gynostemium—frontal view. (**G**) Anther—side view (pollinia and anther cap). (**H**) Friable pollinia pair. (**I**) Labellum—top view. (**J**) Labellum—side view. (**K**) Anther cap—ventral view. (**L**) Bract with papillate margins. (**M**) Dissected perianth, flattened. (**N**) Lateral sepal. (**O**) Papillate margins of the lateral sepals. (**P**) Lateral petal. (**Q)** Trichomes on the stem. (**R**) Lateral sepal—side view. Illustration and photos by Nora E. Anghelescu from the holotype, 21 July 2022 BNP, Romania.

**Figure 4:**
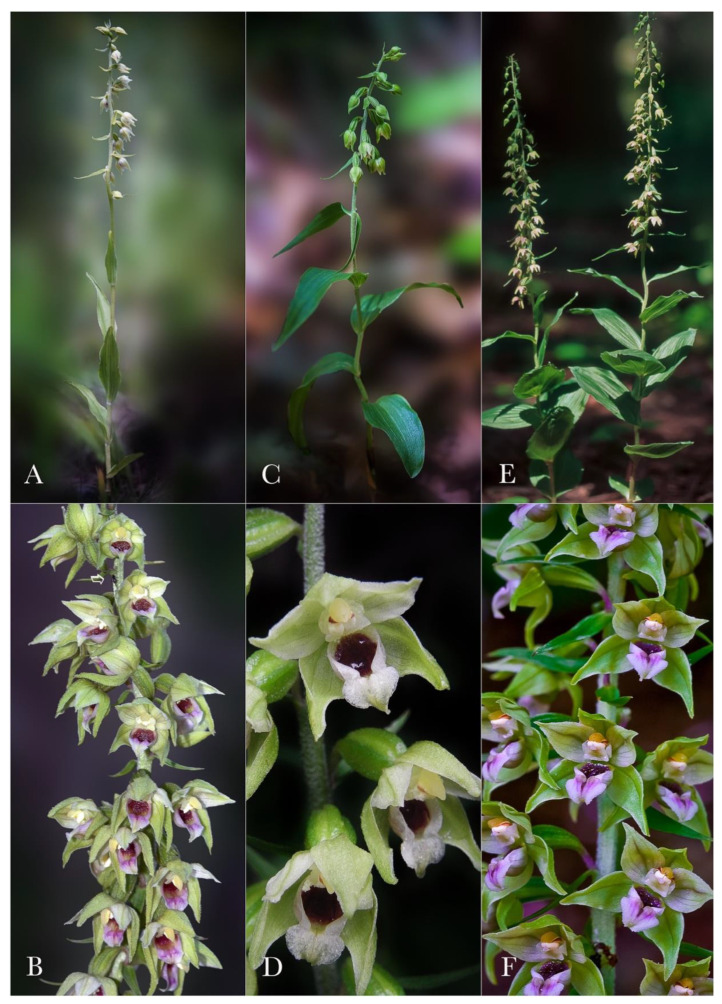
Species comparison: habitus and inflorescence details. (**A**,**B**) *Epipactis bucegensis* (B—flowers hand-opened to show the flower morphology) with shorter, lanceolate, acuminate, yellowish-green leaves (young individual); white (top) arrow—small dipterans (Drosophilidae family) visiting the inflorescences, foraging for nectar or floral exudates. (**C**,**D**) *Epipactis muelleri* Godfery with the characteristic elongated, arched leaves. (**E**,**F**) *Epipactis helleborine* (L.) Crantz with wide, roundish–ovoidal deep-green leaves. Photographs by Nora E. Anghelescu ((**A**) 19 July 2022 BNP; (**C**) 16 June 2018 BNP; (**E**,**F**) 27 July 2009 BNP, Romania), Lori Balogh ((**B**) 17 July 2022 BNP, Romania) and Helmut Presser ((**D**) 18 July 2009, Bavaria, Germany).

**Figure 5:**
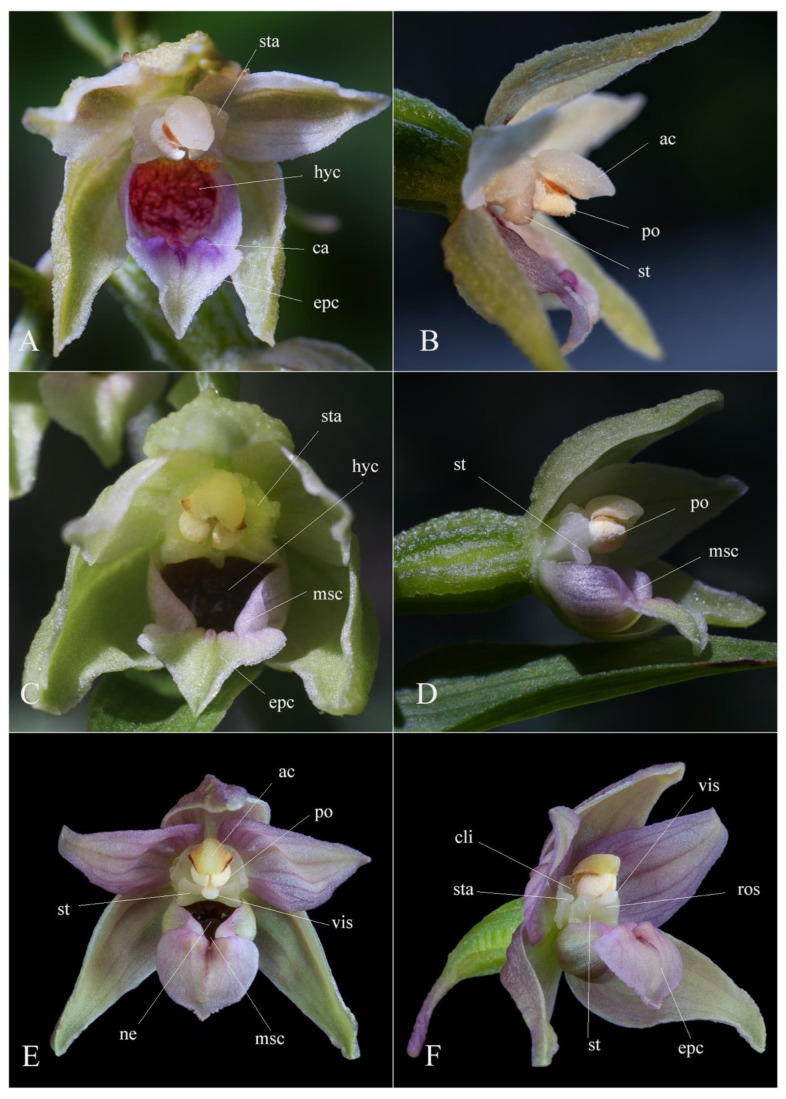
Species flower comparison: frontal and side views. (**A**,**B**) *Epipactis bucegensis* ((**A**,**B**)—flowers hand-opened) without nectar, labellum lacking the mesochile. (**C**,**D**) *Epipactis muelleri* Godfery with tripartite labellum characterised by a well-developed mesochile and faint traces of nectar in the hypochile. (**E**,**F**) *Epipactis helleborine* (L.) Crantz with a nectar-secreting hypochile and a narrow mesochile. Abbreviations: **ac**—anther cap; **ca**—callus; **cli**—clinandrium; **epc**—epichile; **hyc**—hypochile; **msc**—mesochile; **ne**—nectar; **po**—pollinia; **ros**—rostellum; **st**—stigma; **sta**—staminodium; **vis**—viscidium. Photographs by Nora E. Anghelescu ((**A**,**B**) 19 July 2022 BNP; (**E**,**F**) 12 July 2016 BNP, Romania) and Helmut Presser ((**C**,**D**) 20 June 2019, Provence, South-eastern France).

**Figure 6:**
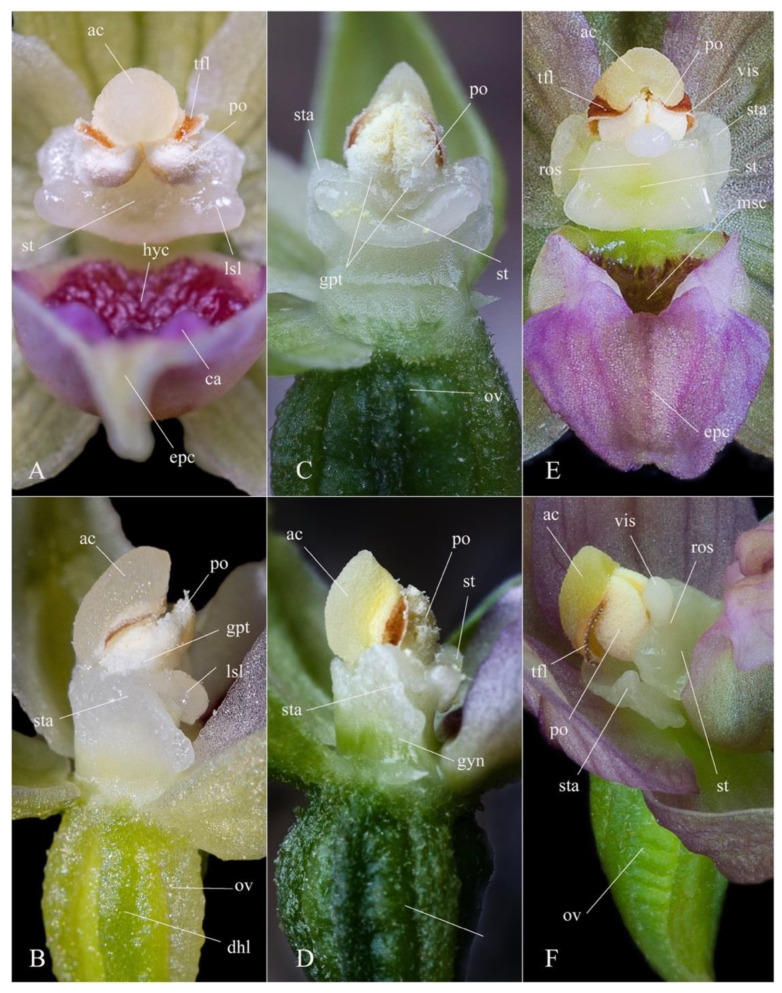
Species comparison: gynostemium (column) frontal and side views. (**A**,**B**) *Epipactis bucegensis*—the anther is pushed forward, facilitating the contact of the pollinia with the stigma; the stigma is rectangular, perpendicular to the gynostemium. (**C**,**D**) *Epipactis muelleri* Godfery—the anther is less angled in rapport to the stigma, but the pollinia still contact the stigmatic surface; the stigma is V-shaped, concave, and narrower. (**E**,**F**) *Epipactis helleborine* (L.) Crantz—the anther is erect and the pollinia are pushed slightly backward due to the presence of the well-developed rostellum and viscidium, which prevent its contact with the stigma. Abbreviations: **ac**—anther cap; **ca**—callus; **dhl**—dehiscence lines; **epc**—epichile; **gpt**—germinating pollen tetrads; **gyn**—gynostemium; **hyc**—hypochile; **lsl**—lateral stigmatic lobe; **msc**—mesochile; **ov**—ovary; **po**—pollinia; **ros**—rostellum; **st**—stigma; **sta**—staminodium; **tfl**—thecal flaps; **vis**—viscidium. Photographs by Nora E. Anghelescu ((**A**,**B**) 19 July 2022 BNP; (**E**,**F**) 12 July 2016 BNP, Romania) and Helmut Presser ((**C**,**D**) 17 July 2016, Rhön, Germany).

**Figure 7:**
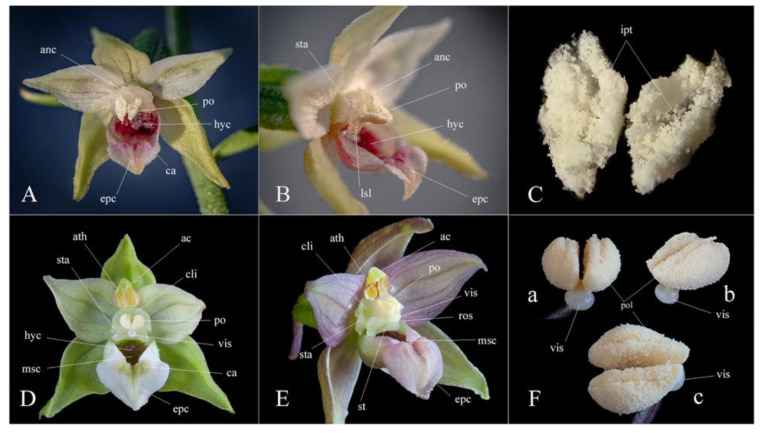
Species comparison: Anther and pollinia details. (**A**–**C**) *Epipactis bucegensis*: (**A**) Anther–top view (without the anther cap), showing the friable pollinia lying freely in the anther due to the total lack of clinandrium. (**B**) Anther—side view showing the pollinia contacting the upper stigmatic surface, with the basal pollen tetrads fixed on the stigma already starting to germinate, enabling self-pollination; note: the anther lacks the viscidium; the stigma lacks the rostellum (upper median lobe). (**C**) Friable, mealy pollinia showing individual pollen tetrads or groups of tetrads disintegrating. (**D**–**F**) *Epipactis helleborine*: (**D**) Anther—top view (with lifted anther cap), showing the compact pair of pollinia resting inside the well-developed clinandrium; **note:** the anther shows a well-developed viscidium. (**E**) Anther—side view showing the pollinia resting inside the clinandrium, well separated by the rostellum, which acts as a barrier between the pollinia and the stigmatic surface, preventing self-pollination. (**F**) Pollinarium composed of a pair of compacted, bipartite pollinia and viscidium attached; pollinarium is removed from the anther as a unit; **a, c.** Pollinarium—top view, showing the pair of pollinia connected to the whitish, spherical, well-developed viscidium adhering to the experimental needle; **b.** Pollinarium—side view. Abbreviations: **ac**—anther cap; **anc**—anther connective; **ath**—anther theca; **ca**—callus; **cli**—clinandrium; **epc**—epichile; **hyc**—hypochile; **ipt**—individual pollen tetrads; **lsl**—lateral stigmatic lobe; **msc**—mesochile; **po**—pollinia; **pol**—pollinarium; **ros**—rostellum; **st**—stigma; **sta**—staminodium; **vis**—viscidium. Photographs by Nora E. Anghelescu ((**A**–**C**) 21 July 2022 BNP; (**D**–**F**) 12 July 2016 BNP, Romania).

Despite the modern molecular techniques, a quick and simple tool to recognize a taxon in field conditions is still required and thus, morphological comparisons prevail in plant identification [23]. Meanwhile, taking into consideration the great phenotypic plasticity of the genus, the macro- and micromorphological features that can be used in taxon delimitation should be carefully assessed. A detailed comparison emphasizing the most significant morphological characters that distinguish *Epipactis bucegensis* from the related species is shown in Table 1 and illustrated in Figure 2, Figure 3, Figure 4, Figure 5, Figure 6 and Figure 7. 

### 2.3. Morphological Distinctness of Epipactis bucegensis 

**Figure 8:**
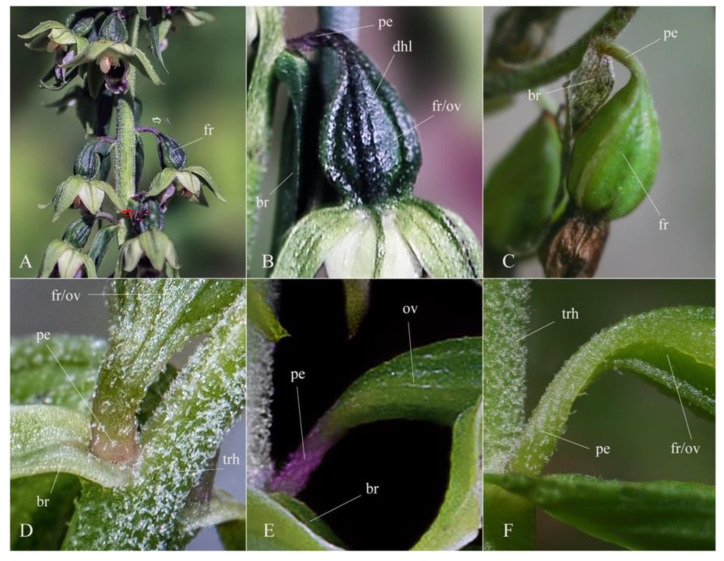
Species comparison: (**A**–**C**) Advanced fruit stages. (**A**) *Epipactis bucegensis* in advanced fruiting stage due to early self-pollination (before anthesis), with flowers hanging on the pendant ovaries; **white arrow**—a juvenile spider female (order Araneae) residing in the inflorescence hunting small flies; **red arrow**—red ants, *Myrmica rubra* (family Formicidae), foraging for food (nectar, floral exudates) on the orchid flowers/inflorescence; (**B**) *Epipactis bucegensis*—close-up of the developing, dark-green fruit with deep, longitudinal ridges (which open in mature, dehiscent seed capsules); the bases of the ovaries are purple-washed; the petioles are strongly purple-pigmented, a sign of increased anthocyanin pigment synthesis. (**C**) *Epipactis muelleri* Godfery—advanced fruiting stages; the fruit is light-green, smooth to faintly ridged. (**D**–**F**) Flower petiole detail—petiole colour plays a major role in the taxonomic identification of *Epipactis* species. (**D**) *Epipactis bucegensis*—petiole showing basal purple pigmentation; (**E**) *Epipactis helleborine* (L.) Crantz—petiole showing deep-purple pigmented base, a major key/typical feature of the species. (**F**) *Epipactis muelleri* Godfery—light-green petiole, showing no purple pigmentation. Abbreviations: **br**—bract; **dhl**—dehiscence lines; **fr**—fruit; **ov**—ovary; **pe**—petiole; **trh**—trichomes. Photographs by Nora E. Anghelescu ((**A**,**B**) 26 July 2009 BNP; (**D**) 19 July 2022 BNP; (**E**) 12 July 2016 BNP, Romania) and Helmut Presser ((**E**) 09 August 2016, Escuaín, Aragonese Pyrenees, Spain; (**F**) 20 June 2019, Provence, South-eastern France).

*Epipactis bucegensis* is morphologically comparable to the autogamous *Epipactis muelleri* Godfery and the allogamous *Epipactis helleborine* (L.) Crantz, but significantly differs from these species in several main characteristics of the vegetal and floral parts (Figure 5, Figure 6, Figure 7 and Figure 8). 

The lowermost leaf size and shape are species-specific features and thus pivotal in species delimitation/separation within the *Epipactis* genus [5,7,9]. As such, the lanceolate–elongated, tapering *Epipactis bucegensis* basal leaf, different from the characteristic roundish–oval basal leaf of *Epipactis helleborine* (L.) Crantz, clearly differentiates/separates the two taxa as separate species. *Epipactis bucegensis* leaves’ distribution on the stem (phyllotaxis) is mainly basal, very different from the middle-stem distribution characteristic to *Epipactis muelleri* Godfery and *Epipactis helleborine* (L.) Crantz. The leaf posture is spreading to erect, sheathing to a subtended angle of ca. 30° relative to the stem, differentiating it from *Epipactis muelleri* Godfery, in which the elongated, arched leaves spread horizontally, curving downwards. The leaf shape is elongate–lanceolate, acuminate, tapering at the tip, vs. the ovoid–elongate, acuminate leaves of *Epipactis muelleri* Godfery and the broadly ovoid to ovoid–elongated, horizontally spread leaves of *Epipactis helleborine* (L.) Crantz. 

The leaf colour, especially in young individuals, is yellowish to yellowish-green, different from the light- to deep-green leaves of the compared species (Figure 2B and Figure 4A,C,E). The colour of the sepals and petals is white to whitish-yellow, vs. the whitish-green to greenish-yellow tepals of *Epipactis muelleri* Godfery (Figure 2A–E, Figure 4B, Figure 5A and Figure 7A,B).

*Epipactis bucegensis*’s unique labellum structure represents its main distinctive feature, making it easily distinguishable, not only from *Epipactis muelleri* Godfery, but from all other European *Epipactis* species (Figure 2D,E, Figure 3A,F,I,M, Figure 5A and Figure 7A). Specifically, the labellum is bipartite, formed of only two parts, the hypochile and epichile, with a completely absent mesochile. By comparison, *Epipactis muelleri* Godfery and *Epipactis helleborine* (L.) Crantz have tripartite labella, with a well-defined mesochile, the narrow junction between the hypochile and epichile. The complete absence of the mesochile is the most distinctive feature of the species (Figure 2D,E, Figure 3I,M, Figure 4A,C,E and Figure 7A,B,D,E). The hypochile shape is characteristically wide, ovoid and flattened, vs. the orbicular, cup-like, deep labellum of *Epipactis muelleri* Godfery. The hypochile inner wall is shiny, dry, crimson-purple-coloured and unusually wrinkled, completely different from that of *Epipactis muelleri* Godfery, which is deep, cup-shaped, roundish, shiny, smooth, blackish-brown and mildly nectar-secreting. The epichile is reduced, triangular, flat, smooth and tapering, vs. the wide–obtuse, deeply wrinkled epichile of *Epipactis muelleri* Godfery and *Epipactis helleborine* (L.) Crantz. Its colour is invariably bright white vs. the greenish-yellow epichile of *Epipactis muelleri* Godfery. The two basal calli found at the base of the epichile are also highly specific, pyramid-shaped, tooth-like, prominent, wide apart, crimson-purple-coloured and smooth/non-wrinkled, vs. the significantly wrinkled and attenuated, greenish-yellow (very rarely pale-pinkish-washed) calli of *Epipactis muelleri* Godfery (Figure 3I, Figure 4B, Figure 5A and Figure 7A,B). 

The gynostemium (column) is specific, with the anther significantly angled relative to the stigma (typical of an obligate autogamous species), differentiating it from the erect gynostemium of the allogamous *Epipactis helleborine* (L.) Crantz. The stigma shape is rectangular, wider than long, bilobed, roof-like and entirely flat, vs. the quadrangular, bilobed, deeply V-shaped and concave one in *Epipactis muelleri* Godfery. 

*Epipactis bucegensis* can be distinguished from *Epipactis helleborine* (L.) Crantz by its modified anther morphology associated with its pollination strategy, obligate autogamy (Figure 6, Figure 7 and Figure 8). The rostellum and viscidium are completely absent, which distinguishes *Epipactis bucegensis* from the allogamous *Epipactis helleborine* (L.) Crantz, in which the rostellum and viscidium structures are well-developed and functional (Figure 5A,B,E,F and Figure 6A,B,E,F). The clinandrium is absent, the highly friable pollinia lying free in the anther, crumbling onto the upper part of the stigmatic surface, vs. the more compact pollinia of *Epipactis helleborine* (L.) Crantz, enclosed in the clinandrium and well-separated from the stigmatic cavity by the roof-like rostellum (Figure 5A,B,E,F and Figure 6A,B,D,E).

The purple-pigmented flower pedicel base of *Epipactis bucegensis* clearly distinguishes it as a separate species from *Epipactis muelleri* Godfery, in which the pedicels’ bases are yellowish to light-green (Figure 8A–D,F). The pedicel-base pigmentation is an essential morphological feature (key) in *Epipactis* species delimitation/separation [5,7]. The fruit of *Epipactis bucegensis* is also specific, highly distinct from *Epipactis muelleri* Godfery. In mature stages, it is pear-shaped, dark-green, purple-washed and strongly ridged on the surface, vs. the elongated, light-green, smoother-surfaced fruit of *Epipactis muelleri* Godfery (Figure 8A,B). *Epipactis bucegensis* was also closely compared to the European autogamous species described in detail in the comprehensive, abundantly illustrated database of the *Arbeitskreis Heimische Orchideen Bayern* e.V. [10], but no similar taxon was observed. The most important feature that distinguishes *Epipactis bucegensis* from all other European *Epipactis* taxa is the bipartite, wide labellum that totally lacks the mesochile (Figure 3I, Figure 4B, Figure 5A and Figure 7A). Therefore, given the significant morphological distinction, its reproductive isolation and its consistent establishment in Bucegi Natural Park, we consider *Epipactis bucegensis* to be a separate (obligate) autogamous species within the *Epipactis helleborine* alliance. 

### 2.4. Morphological Changes to Autogamy

Orchids of the genus *Epipactis* that transition from allogamy to autogamy have to go through various overall morphological changes. To enable autogamy, the pollen should be able to reach the stigma. This is achieved by various adaptations of the flower morphology [30]. The transition from chasmogamous to cleistogamous flowers and some modifications in the architecture of the gynostemium and pollinia structure enable the flowers to switch the pollination strategy from allogamy to (near-) obligate autogamy [31,32]. Allogamous *Epipactis* species attract their specific pollinators with several floral signals, such as flower shape, coloration and complex floral scents (floral volatiles), and reward them with copious amounts of nectar [33]. Nectar is mainly secreted in the concave basal part of the labellum, known as the hypochile (Figure 4D, Figure 5E and Figure 7D). The transition from allogamy to autogamy/cleistogamy is regarded as a more efficient way for the plant to use its energetic/nutritional resources [34].

*Epipactis bucegensis* is an obligate autogamous species that does not require the presence of pollinators, showing all the particular morphological transformations of a typical selfing species. Its flowers are cleistogamous, pendant, scentless and inconspicuously coloured. By blocking the anthocyanin pigment synthesis, the flowers become whitish-creamy to yellowish-green, perfectly camouflaging the plant against the brownish-greenish background of the hot summer, sun-burnt, grassy vegetation characteristic of its preferred habitat (Figure 2 and Figure 4A,B; note: in Figure 2D,E, for the purpose of this study, some of the flowers were hand/manually opened to clearly show the morphology of the floral parts). 

However, despite being an obligate autogamous species, *Epipactis bucegensis* has not lost the ability to produce faint traces of floral nectar (Figure 2A–D, Figure 3B,F,I,J,M, Figure 4B, Figure 5A and Figure 7A,B). The finding was rather surprising since orchids commonly use nectar to attract their pollinators. We found only minute droplets of nectar that accumulated inside the hypochile of the one to two topmost, young flowers (Figure 2E). Minute nectar production was reported several times in other autogams, such as *Epipactis albensis* Nováková and Rydlo [17,33,35], *Epipactis muelleri* Godfery [11] and *Epipactis leptochila* (Godfery) Godfery [30]. These obligate autogams are relatively young species that recently diverged from within the evolutionarily active *Epipactis helleborine* alliance [36]. Moreover, recent studies showed that the chemical composition of *Epipactis albensis* Nováková and Rydlo nectar and scent is partially similar to those of the closely related allogamous species *Epipactis helleborine* (L.) Crantz, further proving its evolutionary origin [33]. The above examples constitute indicative examples of species that transition from ancestral allogamous, insect-pollinating species to obligate autogamy. While still retaining some early features, such as nectar and scent production, these orchids became obligate autogamous/cleistogamous, making insects’ visits nearly impossible [30]. The synthesis of floral attractants or stimuli, i.e., olfactive (scent, odours), food (nectar, food bodies, exudates) and visual stimuli (pigments, colours, shapes, sizes), is highly energy-costly for the plants [37,38]. Once their production is terminated/ceased, the spared nutrients are used by the plants to produce higher numbers of mature, fertile seeds, crucial for their survival and proliferation, a stage regarded as particularly difficult for newly emerged taxa (such as *Epipactis bucegensis*) in the full process of colonising new, nutrient-poor niches [39].

Further, the gynostemium also suffered several morphological transformations. Similar to other autogams, the gynostemium of *Epipactis bucegensis* completely lost the apical structure of the column, termed the clinandrium or anther-bed (Figure 6A,B and Figure 7C,D) an indicative characteristic of autogamous species [40]. In allogamous species, this spacious, hollow structure, situated above the stigma, houses the pair of pollinia, preventing the pollen tetrads from falling off the anther (Figure 5E,F, Figure 6E,F and Figure 7A,B). At dehiscence, due to the lack of the clinandrium, the pollinia, which lay freely in the anther, are projected forwards, falling onto the underlying stigmatic cavity (Figure 3B,C,E–G, Figure 5B, Figure 6A,B and Figure 7A,B). The sessile anther angles even more relative to the stigma, further inclining the pollinia, which can thus easily contact the stigmatic surface (Figure 3C,G, Figure 5B, Figure 6B and Figure 7A,B). The same pollination strategy is employed by other autogams, e.g., *Epipactis muelleri* Godfery (Figure 4C,D, Figure 5C,D and Figure 6C,D).

Additionally, the pollinia of *Epipactis bucegensis* gradually lost coherence and became more friable (Figure 3H, Figure 5B, Figure 6A,B and Figure 7C), disintegrating into individual tetrads or groups of tetrads [28,39]. More compact pollinia, e.g., the pollinia of *Epipactis helleborine* (L.) Crantz (Figure 5E,F, Figure 6E,F and Figure 7D,F), prevent the pollen from falling on the stigmatic cavity [41]. When the pollinia are less coherent, the pollen grains crumble on the stigmatic surface, enabling rapid self-pollination [42]. In the case of *Epipactis bucegensis*, the friability of pollinia is also environmentally dependent. Quite often, external factors, such as high temperatures, humidity and air currents, were reported to influence their friability [43]. In Romania, in July, the outside temperatures may reach 38–40 °C, which causes the pollinia to expand and become even more friable. Apart from the external factors, the flowers hang on fairly long and flexible pedicels, very sensitive to any externally generated movements, such as wind or water drops, which may swing the flowers in all directions. Such movements further increase the disintegration of the mealy pollinia, which crumble onto the viscous stigmatic cavity, situated just below the anther. 

Selfing in *Epipactis bucegensis* is also efficiently promoted by the complete lack of a rostellum, the swollen apical part of the median stigmatic lobe [44], which is well-developed in allogamous *Epipactis* species (Figure 5F, Figure 6E,F and Figure 7E). According to Uphof (1968), ‘*a characteristic of the cleistogamic orchid flower is a very rudimentary rostellum or its absence’* [45]. In allogamous *Epipactis* taxa, a well-developed rostellum creates the most important physical barrier between the male/pollinia and female/stigma parts of the flower, preventing self-fertilization [2,46,47]. In most self-pollinated orchids, however, this structure either does not develop, as in *Epipactis bucegensis* (Figure 6A,B), or, as in *Epipactis muelleri* Godfery (Figure 5C,D and Figure 6C,D) it develops incompletely or sometimes disintegrates during flowering [48]. An important feature in autogams is that, in the absence of the rostellum, the stigmatic cavity usually becomes more active and hypersecreting, being covered in abundant, viscous stigmatic exudate. This is easily observed in *Epipactis bucegensis*, in which the stigma and, in particular, the lateral prominent stigmatic lobes are heavily loaded with viscous, translucid stigmatic exudate (Figure 2E). Just after the impregnation of the pollen grains with the stigmatic secretions, the pollen tetrads start to germinate, producing elongated tubes that grow, fertilizing the ovules (Figure 5B, Figure 6A–D and Figure 7A,B). The pollinia are thus fixed in the anther, immobile, continuously shedding tetrads, a feature that can be observed in many autogamous species [49].

Robatsch (1983) estimated that 60% of *Epipactis* orchids are autogamous, characterized by having powdery pollen that falls onto the stigma [50,51] due to degeneration of the rostellum and relatively low nectar and odour production. In cross-pollinated species, the tip of the rostellum produces adhesive substances, forming a viscidium [52]. In allogamous, insect-dependent *Epipactis* orchids, the viscidium is a protruding sphere-like extension composed of a milky, adhesive liquid, surrounded by a viscidial membrane (Figure 5E,F, Figure 6E,F, Figure 7D–F(a–c)), which connects the viscidium to the pollinarium [36,44]. The main role of the viscidium is to adhere to the pollinators’ bodies and dislodge the pollinia from the anther during pollination (Figure 7F(a,b)). The presence of a large, viscous viscidium ensures that the pollinia are removed by pollinators and hence, the level of autogamy is decreased. 

### 2.5. Pollination Monitoring

True *Epipactis* pollinators are usually large, strong insects capable of carrying the heavy load of pollinia. Our observations included various hymenopterans—wasps (family Vespidae), bees (family Apidae), bumblebees (mainly genus *Bombus*) and ants (family Formicidae); coleopterans—beetles (Cerambycidae and Oedemeridae families); and large dipterans—forest flies (family Anthomyiidae). They usually feed on copious amounts of nectar secreted by allogamous *Epipactis* species such as *Epipactis helleborine* (L.) Crantz*, Epipactis purpurata* Sm.*, Epipactis distans* Arv.-Touv. and *Epipactis atrorubens* (Hoffm.) Besser [19]. In *Epipactis bucegensis*, the viscidium is not formed as a consequence of the absence of the rostellum (Figure 6A,B and Figure 8B). Similarly, the rostellum is absent in *Epipactis muelleri* Godfery (Figure 5D and Figure 6C,D). Thus, the complete lack of the rostellum–viscidium structure(s), accompanied by the friable pollinia and hypersecreting stigma, resulted in very efficient self-pollination, consequently reducing the chances of pollen being transported by insects. As a result, the cleistogamous flowers of *Epipactis bucegensis* self-pollinate during the early stages or even before anthesis (in the bud stages). This was confirmed by the fact that, during the 10–12 days of field research, we did not observe any true pollinating insects visiting the flowers of *Epipactis bucegensis*. Nevertheless, the flowers were accidentally visited only by sporadic small forest flies of the family Drosophilidae (Figure 4B, white arrow) and red ants, *Myrmica rubra* (family Formicidae, Figure 8A, red arrow). These random visitors are only food foragers, searching for nectar or floral exudates during their visits. They are not true orchid pollinators, since they are too small to carry or displace the heavy pollinia from the anther. In one instance, a small female spider (Figure 8A, white arrow) was observed to reside in one of the inflorescences, using it as a hunting site for its small dipteran prey. Spiders (order Araneae) are the most common predators in orchids, found to inhabit the inflorescences of many orchid species, successfully preying on their pollinators [53].

Thus, the inconspicuously coloured, nectarless, scentless, cleistogamous flowers of *Epipactis bucegensis* show all the characteristic features of a typical obligate autogam capable of forming healthy, new populations, completely independent of the presence of pollinating insects. Nevertheless, autogamy is rarely absolute. There is always a chance that an insect of a suitable size, usually a food forager, either a true pollinator or a visitor, occasionally visits the nearly closed (cleistogamous) flowers of *Epipactis bucegensis*. Because the species does not produce a viscidium, even when the flowers are penetrated by insects, the pollinia do not attach to their bodies. Instead, due to the insects’ disturbance and movements, the pollinia disintegrate even more, spreading onto the stigmatic surface, and thus, self-pollinating the flowers.

The early swelling of the ovaries is also a clear indication of early autogamy [54,55,56,57]. Even before the topmost flowers reach maturity, the basal ovaries are already swollen, while still keeping the withered flowers hanging on the capsules. Within 2–5 days, almost all ovaries develop into dark-green, purple-tinted, pear-shaped, swollen fruits (Figure 2A,B and Figure 8A,B). The fruit set is very high, up to 90–98% (in 65 counts), a characteristic of autogamous species. In a few individuals, the upper 1–2 flowers remain non-self-pollinated, being eventually aborted by the plant. Once the fruits start to swell, the initial yellowish-green colour of the ovaries and leaves gradually changes to dark green (Figure 8A,B). This indicates a significant increase in the photosynthetic activity of the plants, which start to produce higher amounts of carbohydrates to accomplish the maturation of the fruits and seeds, thus assuring their successful reproduction and proliferation. Similar quick and efficient self-pollination strategies were observed in other autogamous *Epipactis* species, such as *Epipactis muelleri* Godfery*, Epipactis albensis* Nováková and Rydlo*, Epipactis leptochilla* (Godfery) Godfery and *Epipactis phyllanthes* G.E.Sm. [28,29,36,58]. 

## 3. Discussion

### 3.1. Active Speciation within the Epipactis Genus

*Epipactis* is regarded as an evolutionarily young genus that, recently, has undergone a rapid process of diversification and speciation [23,59], with numerous new (mostly) autogamous species being described. According to Delforge (2006), during the last glaciation, these species had their distribution restricted to the south, to the Iberian, Italian and Balkan peninsulas, as well as the Caucasus. With the amelioration of the climate, which began at around 10,000 B.C.E, the beechwoods moved slowly northwest, reaching Scandinavia at around 500 C.E. This recent arrival in mid-Europe may explain why *Epipactis* seems to be in the process of evolutionary radiation and why the taxonomic treatment of the genus is rather challenging [5] 

Based on extensive phylogenetic analyses, it was suggested that the newly emerged, near-obligate autogams had repeatedly radiated across Europe from within the more widespread, putative universal ancestral species, the predominantly allogamous *Epipactis helleborine* sensu stricto (s.s.). According to Sramkó et al. (2019), *Epipactis helleborine* (L.) Crantz is, most probably, the direct ancestor of at least ten recently derived species, the majority of them near-obligate autogams, such as *Epipactis leptochila* Godfery) Godfery*, Epipactis greuteri* H.Baumann and Künkele, *Epipactis muelleri* Godfery*, Epipactis albensis* Nováková and Rydlo and *Epipactis dunensis* (T.Stephenson and T.A.Stephenson) Godfery. In evolutionary terms, these facultative/near-obligate autogams were supposed to have undergone a fairly recent, rapid separation from their ancestral genetic background [36]. Authentic speciation events can lead to the formation of autogams from allogams, although autogams are believed to constitute evolutionary dead-ends, no autogam ever being able to generate further autogamous species, as reported previously [23,29,60,61,62,63]. Consequently, this excludes the possibility of an eventual radiation/emergence of *Epipactis bucegensis* from obligate autogams, such as *Epipactis muelleri* Godfery. Nevertheless, further detailed phylogenetic analyses are needed to elucidate the potential direct ancestral species of *Epipactis bucegensis*, the time of radiation and its phylogenetic relationships within the aggregate. As such, the *Epipactis helleborine* alliance represents an example of an active evolutionary clade, within which speciation events have occurred comparatively recently, mainly through transitions from allogamy to autogamy [24,26,64].

It is well-known that self-compatible *Epipactis* orchids are well adapted to switch from allogamy to autogamy, depending on the degree of the environmental factors’ adversity, which may accelerate the process [2,36,47]. Thus, the natural pressure imposed by the external factors may accelerate this transition process, causing autogamy to occur with increasingly high frequency in successive flowering seasons, ultimately leading to genetic drift, i.e.*,* the change in the frequency of an existing gene variant (allele) in a population due to random chance [65], also known as *allelic drift* or the *Wright effect* [66]. There are many examples of species that can act as both cross-pollinating (pollinator-dependant) and auto-pollinating, depending on various external factors of their natural habitats. Thus, even in the obligately allogamous species, autogamy was shown to incidentally take place [31,33,67]. Both autogamous and allogamous flowers within the same *Epipactis helleborine* (L.) Crantz plant were reported several times [3,42,50,68,69]. Additionally, it was reported that, as an adaptation to extreme conditions, obligate allogams, such as *Epipactis helleborine* ssp. *neerlandica* (Verm.) Buttler and *Epipactis helleborine* subsp. *orbicularis* (K.Richt.) E.Klein (now *Epipactis distans* Arv.-Touv.), can change their mode of pollination from allogamy towards autogamy [54]. In temperate regions, they are allogamous and well-visited by insects [35,70]. However, in xerophilous regions, they may become facultative autogams even before anthesis [54,55,56,57]. Therefore, the actual pollination syndrome or the reproductive strategy can be significantly influenced by floral ontogeny (age of the flowers), environment (temperature, high or low humidity, drying winds, etc.) or both [30,61,62]. Nevertheless, the evolutionary (morphological) transition from obligate allogams to obligate autogams is the result of a combination of developmental genetic, epigenetic and ecophenotypic factors, as a consequence of both prolonged natural selection pressure and genetic drift [36]. 

It must be mentioned that the evolutionary shift from cross-fertilisation to self-fertilization is one of the most frequent evolutionary transitions in plants. It is believed that autogamy is employed by approximately 10–15% of flowering plants [71] as an adaptation to growing in harsh, unfamiliar habitats where, usually, the specific pollinating insects are lacking [31]. There have also been numerous reports of autogamy in the orchid family [72]. Among the temperate orchids, apart from the *Epipactis* genus, self-pollination (facultative and/or obligate) has been found in several other genera such as *Ophrys* L., *Pseudorchis* Ség., *Neottia* Guett., *Cephalanthera* Rich., *Chamorchis* Rich. and *Corralorhiza* Gagnebin [19,39,73]. The more extreme the conditions in which an orchid grows (biotope, habitat and/or climate changes, presence/absence of pollinators, etc.), the higher the chances that it will turn towards autogamy as a survival strategy. Anthropogenic factors, mainly the destruction and loss of the original habitats (agriculture, urban expansions, deforestation, etc.), leaving only small suitable patches for the orchids, probably also contributed to the switch of pollination mode and reproductive strategy [30]. Regardless of the presence or absence of pollinators, independence from insects offers orchids an opportunity to conquer new habitats, assuring unconditional, certain reproductive success [71,74,75,76]. Shady woodlands with comparatively impoverished ground floras, where pollinator visits are likely to be less frequent, are the preferred habitats of most of the autogams. Hence, the increased ability of self-pollinating orchids to colonise new ecological niches may explain the large geographic area that the newly formed autogamous *Epipactis* species can occupy [36].

### 3.2. Inbreeding—Friend or Foe?

In nature, most plant and animal species have evolved various mechanisms to avoid inbreeding. Inbreeding produces increased homozygosity of recessive partially deleterious mutants and by chance in small populations, such as isolated populations of autogamous plants, these alleles can become fixed [77]. Repetitive autogamy leads to population inbreeding depression, generally expressed by an increased frequency and accumulation of recessive lethal or mildly deleterious mutations. Consequently, the individuals experience significantly reduced viability and fecundity, which ultimately leads to a sudden decline in population numbers [75]. In the early 19th century, Darwin argued that outcrossed offspring of plants are usually fitter and better adapted to survive than those produced by self-fertilization [78,79]. He considered that flowering plants evolved well-adapted features to enable outcrossing, thus avoiding inbreeding depression caused by selfing, as the predominant mode of reproduction [80,81,82].

Despite the commonly believed disadvantages of inbreeding, studies/observations of dominantly allogamous species vs. the dominantly autogamous species within the *Epipactis* section revealed that there is no noticeable deleterious effect of selfing in the recently formed autogams [39]. According to Sramkó et al. (2019), inbreeding depression in *Epipactis* lineages may be either counterbalanced by outbreeding or cleared out from the autogams by natural selection that acts on the unmasked deleterious recessives. At the same time, the average distributional areas or population sizes/counts proved not to be significantly different between the already established allogams and recently radiated autogams. Thus, it was suggested that the great genetic diversity of *Epipactis helleborine* (L.) Crantz, together with its greater phylogenetic range, enabled it to function rather successfully as a source of the future novel (autogamous, cleistogamous) species [36]. 

### 3.3. The Role of Cleistogamy in Active Speciation

In the case of geographically localized populations that suffer genetic isolation from their progenitors, active speciation may take place, generating new lineages, mostly with a tendency towards producing cleistogamous flowers, i.e., flowers that do not open and are self-fertilized in the bud [2,42], a tendency strongly expressed by *Epipactis bucegensis*. Cleistogamy prevents the access of insects, invariably leading to obligate autogamy. Nevertheless, some authors further suggested that this transition in the breeding system was unidirectional, the allogams never arising from autogams, which makes the autogamous *Epipactis* species potentially evolutionary dead-ends [29,60,61,62]. Varying degrees of autogamy were reported in several other groups, e.g., the *Spiranthes sinensis* (Pers.) Ames species complex, in which autogamy has contributed to intraspecific morphological variability and, in some instances, speciation [63].

A typical feature of obligately self-pollinating taxa is that the newly emerged group(s) are highly homogenous, while there are considerable differences between different populations [27,28,29,47]. Squirrell et al. (2002) noted that: ‘*With each generation of complete selfing, homozygosity increases by 50%. In this fashion, a large genetic distance arises rapidly between progenitor and derivative species*’ [28]. This has led to an increase in speciation, mostly represented by local (micro)endemic forms, demonstrating the plasticity of the genus and the dynamics of its evolution [11].

The cleistogamous, micro-endemic *Epipactis bucegensis* may represent an example of a recently genetically separated autogam that eventually colonized new habitats and successfully reproduced and proliferated, independent of the pollinators’ presence. 

Discovered 14 years ago, *Epipactis bucegensis* proved to form stable, large, healthy populations in the south-eastern part of Bucegi Natural Park, at the same time presenting highly preserved specific characters that showed little to no variability. The essential morphological features (keys) in *Epipactis* species separation, such as the creamy-white, pendant, cleistogamous flowers; the unique structure of the labellum lacking the mesochile; the distinctive pyramidal/triangular purple-coloured labellar calli; and the purple-pigmented base of the pedicel and fruit represent species-specific characters, which significantly distinguish it from the related *Epipactis* taxa. 

Therefore, our thorough approach strongly supports the recognition of *Epipactis bucegensis* as a morphologically, phenologically and ecologically distinct species within the *Epipactis helleborine* aggregate.

## 4. Materials and Methods

### 4.1. Sites Studied

The studies were conducted in three subalpine areas within the Bucegi Natural Park, a protected area included within Natura 2000 site ROSCI0013, IUCN category V (Protected Landscape, Law No. 5, 6.03.2000), covering Prahova, Dâmbovița and Brașov Counties, Southern Carpathians, Central Romania, with an area of ca. 32.663 ha/326.63 km^2^ and the highest elevation (elev.) at Omu Peak of 2505–2514 m a.s.l (above sea level).

### 4.2. Populations Counts

The first population of *Epipactis bucegensis*, counting a total of 5–6 individuals, was discovered by NEA on 26 July 2009 in Prahova County, Bucegi Natural Park, elev. 810–960 m a.s.l. Its occurrence was subsequently monitored in 2010 and 2011, counting 3–4 and 6–7 individual plants, respectively. Several digital photographs were taken, but neither detailed measurements nor formal descriptions were performed at the time. Unfortunately, further monitoring of the first *Epipactis bucegensis* population was not possible as the area was destroyed and most of the present flora was lost due to real estate development. Nevertheless, on 17 July 2022, during a botanical field study, two new populations were discovered by LB and MB in the south-eastern part of the park, in Dămbovița County, elev. 820–980 m a.s.l. Together, the two newly discovered populations contained a total of ca. 60–75 individuals (ca. 45–55 and 10–15 individuals/population). The plants were found occurring individually or in groups of 2–6 siblings. The initial population numbers might have been higher since the areas were used as cattle fields and part of the vegetation was already destroyed by the grazing animals. 

### 4.3. Extent of Occurrence (EOO)

The populations were found growing nearby, at a distance of 3–5 km, with an EOO of ca. 10–15 km^2^ each (Figure 1C, red dots).

### 4.4. Species Studied

*Epipactis bucegensis* plants were collected between 17 and 21 July 2022 under the permit granted by the Bucegi Natural Park Administration: Research Permit No.1887/CAN/22.07.2021–2023 APN–Bucegi (RO: Administratia Parcului Natural Bucegi).

### 4.5. Study Time Frames

17–25 July 2022. 

### 4.6. Morphological Comparisons

Measurements of the vegetative and floral parts were made from living plants and fresh flowers. To describe this newly found population as comprehensively as possible, a total of 117 morphological characters were compared, out of which 37 morphological characters were measured directly from living plants and flowers. The morphological characters used for the study included most of the characters used previously [83]. Special attention was given to the characters that proved to be taxonomically informative and those that involve the differentiating details in the morphology of the leaves, gynostemium, labellum, pollinia, ovary and fruit. The measurements are examples of the new taxon, *Epipactis bucegensis,* and its related species, the autogamous *Epipactis muelleri* Godfery and the allogamous *Epipactis helleborine* (L.) Crantz.

### 4.7. Pollination Monitoring

Monitoring was conducted for a total of 4–6 h per day, between 17 and 21 July 2022 when most of the flowers were in full anthesis. Nevertheless, the cleistogamous flowers were never fully opened; hence, pollinator presence/attraction was rather scarce. The observer (NA) was initially located approximately 2–3 m from the subjects (groups or individual plants). Once various insects were observed to patrol and/or approach the flowers, they were (intended) to be recorded in digital photographs (note: no insects were collected or harmed in any way during the study).

### 4.8. Digital Photographic Equipment

Digital images of individual plants and floral parts were taken using Nikon D3 and Nikon D850 camera bodies equipped with Nikon Micro NIKKOR 60 mm and NIKKOR 24.0–70.0 mm lenses. Additional equipment included a Manfrotto Tripod and Litra Torches 2.0s. An adapted Helion FB tube was used for automated focus bracketing. The images were analysed using Adobe Photoshop^®^ CC 2023, Zerene Stacker Software, Vers.2021-11-16 [84]. 

### 4.9. Maps

The map was created using ArcGIS Pro 3.1 software; the maps and elevation services were provided by the entities mentioned in the copyright.

## 5. Conclusions

Autogamy is a common reproductive mechanism used by many species of flowering plants, including the complex orchid genus *Epipactis*, as an adaptation to colonise new habitats. This monophyletic clade, with numerous, mostly newly evolved autogamous species is presently undergoing evolutionary radiation driven by a large spectrum of genotypic (genetic and/or epigenetic factors, genetic drift), phenotypic (ecophenotypic) and environmental factors (habitat changes, climate changes, presence/absence of true pollinators and specific mycorrhizae). Ancestor species, such as *Epipactis helleborine* s.l., have been shown to have, rather frequently and recently, generated many isolated, local autogamous (often cleistogamous) forms. These, generally viewed as examples of incipient speciation from within the parental genetic background, are often as widespread and ecologically successful as allogams, a result of a high level of initial/incipient genetic variation [29], which gives them the potential to evolve into new taxa [36].

Thus, due to the great phenotypic plasticity of the genus in response to environmental requirements, the formation of micro-endemic populations with different reproductive mechanisms led, in recent years, to noticeable, fast changes within the taxonomy of the *Epipactis* genus [85]. Novel morphological adaptations to new, isolated habitats are constantly described, often making the recently emerged taxa the subject of much discussion [60,86] and *Epipactis* one of the most taxonomically complex and dynamic orchid genera in Europe. 

## 6. Taxonomic Treatment

***Epipactis bucegensis* N.Anghelescu, L.Balogh and M.Balogh, sp. nov.** (Figure 2, Figure 3, Figure 4, Figure 5, Figure 6, Figure 7 and Figure 8).

**Holotype:** Romania. Southern Carpathians, Bucegi Natural Park ROSCI001 Natura 2000: Șipotului Valley of lower Dămbovița County, grassland, deciduous to mixed forest, calcareous conglomerate, leg. Nora E. Anghelescu sub No. 1–2 ex. specimen typorum: GPS: 45°17′33.95″ N; 25°30′26.41″ E, elev. 750–890 m a.s.l., 10–30 July 2022, fl. 15–17 July 2022, deposited at the Herbarium of the Botanical Garden Bucharest *NE Anghelescu NEABUC410357* (Holotype: BUC!). Additionally, the holotype was confined to printed digital images: 120 images by LB and MB on 17 July 2022 and ca. 900–1000 images by NEA between 19 and 21 July 2022, deposited in private image databases. 

**Diagnosis:** *Epipactis bucegensis* is most morphologically similar to *Epipactis muelleri* Godfery but can be distinguished by its elongate–lanceolate, near erect, yellowish leaves; creamy-white to whitish-yellow petals and sepals; its wide, ovoidal labellum that completely lacks the mesochile (the narrow, middle segment); and its purple-pigmented petiole base and purple-washed mature fruit.

**Description:** Described exclusively from living plants and flowers.

Terrestrial, perennial, rhizomatous, autotrophic, sympodial herbaceous geophyte, 18–25(40) cm tall, including inflorescence. ***Rhizome*** (hypogeal) 4–12(16) cm long, 1.3–2.5(3.3) cm diam., branchy, thickened, creeping, horizontal. ***Roots*** 2(3.4)–10(12.4) cm long, 1.5–2.0(3.2) mm diam., adventitious, cylindrical, fleshy, thick, numerous, with no tubers. ***Stem*** (epigeal) 18–30(35) cm, 0.3–0.5(0.8) cm diam., erect, spindly to flexuous, yellowish-green, completely devoid of purple pigmentation (anthocyanins), glandular–pubescent (densely covered in whitish glandular hairs, trichomes), especially in the upper and basal parts. ***Trichomes*** dense, glandular, whitish-translucent, covering the stem and flower pedicels (Figure 1). ***Basal sheath*** 1.5–2.5 × 0.9–2.0 cm, green, unspotted, sessile, narrowly elongated, widest at the base, keeled, acuminate/tapering at the tip, with apical hooding. ***Cauline leaves*** 5–8(10), yellowish-green turning to deep-green with age, unspotted, sessile, sheathing along the length of the stem, placed in two opposite rows (alternate/spirally arranged), oval–lanceolate, widest in the middle, keeled, deeply veined with distinct midrib, tapering/acuminate, partially arched and relatively stiff, longer than the stem internodes, with slightly undulate margins. Leaves and bracts edged with fine, hyaline/translucent, conical, rather irregular tooth-like papillae/serrations, forming single-row clusters or occurring separately, even at the same leaf margin, with a tendency to group in the undulated sectors. ***Basal/median******cauline leaves*** 3–5(8), 5.2–10(12) × 1.5–6.0 cm, basal leaves may be shorter but wider than the longest leaf, yellowish-green, oval–lanceolate, tapering, longer than internodes, spreading, near-erect, to a subtended angle of c. 35°–40° relative to the stem. ***Upper******cauline leaves*** 1–2(4), 3.0–4.4 × 0.5–1.0 cm bract-like, yellowish, near erect, narrow–lanceolate. Large ***gap***, 2.0(4.2)–5.1 cm, present between the uppermost leaf and the base of the inflorescence. ***Lower flower bracts*** 15–38(45) × 3.2(4.2)–8.0(12) mm, leaf-like, near horizontal, lanceolate, acuminate, significantly longer than the flowers, robust, keeled, horizontally spreading to a subtended angle of c. 90˚ relative to the stem (perpendicular to the stem). ***Upper flower bracts*** 1.8–2.2 × 0.2(0.3)–0.4(0.8) cm, longer than the ovary, shorter than the flowers, membranous to robust, horizontally spreading to a subtended angle of c. 90° relative to the stem (perpendicular to the stem). ***Inflorescence*** 10–15 × 3.5–4.5(5.5) cm (rachis length × width of inflorescence calculated from bract-tip to bract-tip), terminal, lax to dense, elongated raceme, near-one-sided, floriferous, acropetal (opening from the base upwards). ***Flowers*** 10–30(50/65), 8.5–11.2 × 4.5–6.5 (diam.) mm, medium-sized, unscented/scentless, pendent, bell-shaped, inconspicuously coloured in shades of whitish-yellow to yellowish-greenish, resupinated, usually closed, rarely partially opened (cleistogamous), rapidly withering. ***Tepals*** free, converging, arranged into a campanulate perianth, similarly whitish-yellow to pale-greenish colored; inner faces whitish to yellowish-green, tapering towards the apex, acuminate, slightly concave, nerved, with prominent median nerve, glabrous, dorsally/abaxially papillose. ***Sepals*** 3, 7.0–14.2 × 5.5–7.1 mm, yellowish, washed green, deeply/distinctly median keeled, ovate to elongated ovate, widest in the basal half, with elongated acuminate apexes, 4–6 nerved, glabrous, with a papillose outer/adaxial surface, edged with fine, hyaline, rather irregular tooth-like serrations, up to 0.1–0.2(3) mm high, forming single-row clusters on both margins towards the apex. Median/dorsal sepal and lateral sepals nearly similar in colour (yellowish-green) and shape (ovate) and equal in size. ***Petals*** 2 lateral, 6.0–9.0 × 4.0–6.1 mm, similar to sepals but slightly smaller/shorter, broadly ovate in outline, whitish-yellow, keeled, centrally washed green, with whitish lateral margins, widest in the basal half, tapering towards the apex, rather concave, widest in the middle, 3–5 nerved, glabrous, with papillose outer/adaxial surface, margins entire or (rarely) edged with fine, hyaline, rather irregular tooth-like serrations, up to 0.1–0.2 mm high, forming single-row clusters on both margins towards the tapering apex. ***Labellum*** glabrous, split in only two joined segments: hypochile (basal segment) and epichile (apical segment). ***Hypochile*** 3.0–4.1 × 4.0–5.2 mm, cup-shaped, concave, outer rim white to washed pink, shiny, with purple- to dark-purple-coloured inner surface; external/adaxial surface of the cup whitish to pale-pink, scarcely nectar secreting. ***Nectar*** absent in older flowers, present in minute amounts/faint traces exclusively in (1–2) young topmost flowers. ***Mesochile*** junction completely absent, the hypochile and epichile forming a continuous body. ***Epichile*** 5.0–5.8 × 3.0–4.2 mm, triangular, non-hinged, flat to slightly convex, whitish-yellow, entire margins, linguliform, spreading, mildly three-lobed; lateral lobes small to almost absent, scalloped, whitish, median lobe triangular, with acuminate elongated, near-straight to pointing down apex, presenting two lateral, tooth-like, pyramidal/conical, purple/crimson, non-wrinkled basal calli, widely separated by a short, roundish, minute central callus followed by a median/longitudinal groove. ***Spur*** absent. ***Gynostemium*** (column) 1.5–2.6 × 1.8–2.1 mm, short, erect, cylindrical, translucent white, with a wide base, forward-projecting above the hypochile. ***Staminodes*** present, wing-like, formed on each side of the gynostemium, laterally (sub)flanking the stigmatic cavity. ***Auricles*** absent. ***Clinandrium*** (anther/pollinia bed) absent, facilitating the direct contact of the pollinia with the stigma. ***Anther*** 1.1–1.9 × 1.3–1.6 mm, translucent white to creamy-white, short, ovoid, thick, whitish, bithecal (with two anther cells), pushed-forward, sessile, broad, dehiscent before anthesis. ***Pollinia*** 1.5–2.3 × 0.5–1.2 mm, two, lacking caudicles, clavate, ovate–elongated, whitish to creamy-white, powdery/mealy, very friable, crumbling and disintegrating on the upper margin/rim of the stigmatic surface, leading to autogamy. ***Pollen*** composed of many pollen grains arranged in isolated tetrads, loosely connected. ***Viscidium*** absent, facilitating self-pollination. ***Anther cap*** 2.0–2.5(2.9) × 0.9–2.2 mm, pale yellow, well-developed, thick, ovate–elongated to ovoidal, papillose, with shiny outer surface and lateral, brownish-orange flaps, reminiscent of the two dehiscent anther thecae. ***Stigma*** 1.6–1.8 × 2.1–2.3 mm, placed just below the anther/pollinia, quadrangular, perpendicular to the axis of the gynostemium, flat, board/roof-like, large, bilobed (rostellum, the median lobe absent), rectangular, wider than long, with prominent, lateral, convex stigmatic lateral lobes flanking a mildly concave, viscous, sticky central stigmatic cavity with prominent lower rim. Stigmatic surface and lateral stigmatic lobes covered in abundant viscous, stigmatic secretions/fluid. ***Rostellum*** absent. ***Ovary*** 6.2–8.5 × 3.9–4.8 mm pendant, pear-shaped (clavate), slightly curved but not twisted, thick, minute, scarcely papillose, with downy outer surface, yellowish-green to dark-green (especially in the fruiting stages), unilocular with parietal placentation and well-marked, six longitudinal ridges (three parietal placentation, three dehiscence ridges). ***Flower pedicel*** 1.2–2.5 × 1.0–1.2 mm downcurved, short and thick, yellowish-green, with very faint traces of basal anthocyanins, mostly unpigmented, mildly downy/pubescent, covered in whitish glandular hairs (trichomes), not twisted, with a pale-greenish base. ***Fruit*** 6.9–10.5(12.3) × 4.9–5.9(6.3) mm, deep- to dark-green, washed purple at the base, roundish–ovoid, wide at apex, pendant capsule, with six highly pronounced longitudinal ridges, fruiting end-July–August, fruit set 75–93% in 65 counts. ***Seed capsule*** 7.1–12(13.1) × 5.1–6.5 mm single, brownish capsule, larger than the fruit pod, with three (opened) dehiscence ridges. ***Seeds*** ca. 0.94–0.29 × 0.2–0.03 mm, minute, numerous, narrowly obovate, tapering from the middle to the tips, with reticulate–foveate ribs/folds on the testa cells; maturation time September–October.

**Cytogenetics:** Chromosome numbers are very variable within the genus, with a basic chromosome number x = 10 [84]. The species might be similar in chromosome number to its relative *Epipactis muelleri* Godfery 2n = 4 [5]; nevertheless, this still needs to be determined.

**Flowering period:** The species has been observed exclusively in its natural habitat flowering from the beginning to mid-July. The flowers’ longevity is very short to absent, self-pollination/autogamy occurring before the anther dehiscence, while still in the bud stages (cleistogamy). Nevertheless, we noticed closed flowers still hanging on the developing/swollen fruit capsules for several days before showing clear signs of flower senescence (flower wilting or shedding of the floral parts). 

**Habitat:** *Epipactis bucegensis* prefers a cool subalpine climate, with moderate humidity, in full sun to partial shade, on dry to moist, neutral to calcareous/alkaline substrates. It also grows in open woodland, next to forest edges, in mixed (deciduous and coniferous) forests, grasslands, shrublands and anthropogenic habitats, such as rural and urban roadsides, lawns or private estates. 

**Ecology:** Individuals of the species have been found occurring either as isolated adult plants, separated by a distance of ca. 10–30 m, or aggregated, forming small- to medium-sized groups (usually n < 10) composed of several siblings and one to three adult plants. Our field observations suggest that plants usually synchronize their blooming, most of them flowering during the hottest summer season, which usually corresponds to the months of July–August (in temperate Romania/Europe). The dominant tree species found growing in the vicinity of *Epipactis bucegensis* were common woody species such as *Abies* Mill. spp. (fir, coniferous, family Pinaceae), *Acer platanoides* L. (Norway maple, family Sapindaceae), *Juniperus communis* L. (common juniper, family Cupressaceae), *Fagus sylvatica* L. (European beech, family Fagaceae), *Elaeagnus rhamnoides* (L.) A.Nelson (sea-buckthorn, family Elaeagnaceae) and *Sorbus aucuparia* Poir. (rowan, family Rosaceae). Herbaceous species include *Achillea millefolium* L. (yarrow, family Asteraceae), *Taraxacum campylodes* G.E.Haglund (common dandelion, family Asteraceae), *Centaurea* L. spp. (centaury, family Asteraceae), *Knautia longifolia* (Waldst. and Kit.) W.D.J.Koch (widow flower, family Caprifoliaceae) and *Leucanthemum vulgare* (Vaill.) Lam. (ox-eye daisy, family Asteraceae). Orchid species found to occur sympatrically at a similar elevation, phenologically active from May to August, include *Anacamptis morio* (L.) R.M.Bateman, Pridgeon and M.W.Chase, *Dactylorhiza fuchsii* (Druce) Soó, *Dactylorhiza fuchsii* subsp. *carpatica* (Batoušek and Kreutz) Kreutz, *Dactylorhiza incarnata* (L.) Soó, *Dactylorhiza saccifera* (Brongn.) Soó*, Coeloglossum viride* (L.) Hartm*., Gymnadenia conopsea* (L.) R.Br*., Orchis mascula* (L.) L. subsp. *speciosa* (Mutel) Hegi*, Orchis militaris* L.*, Neottia ovata* (L.) Bluff and Fingerh*., Neottia nidus-avis* (L.) Rich.*, Platanthera bifolia* (L.) Rich*., Cephalanthera rubra* (L.) Rich., *Cephalanthera damasonium* (Mill.) Druce*, Epipactis palustris* (L.) Crantz, *Epipactis helleborine* (L.) Crantz, *Epipactis microphylla* (Ehrh.) Sw. and *Epipactis exilis* P.Delforge. 

**Variability:*** Epipactis bucegensis* individuals do not show any significant visible variation apart from slight differences in the height of the plants and, consequently, the number of flowers in the inflorescences. Nevertheless, this may be due to different plant ages, siblings being usually shorter than the parental individuals with fewer flowers. However, most floral characters do not show significant variability, the whitish-yellow colour of the cleistogamous flowers and their large numbers/inflorescence being rather constant in the population. 

**Locus classicus:*** Epipactis bucegensis* is endemic to the restricted original (holotype) geographic area located on the south-eastern side of Bucegi Mountains Natural Park ROSCI001 protected area Natura 2000, IUCN category V (Protected Landscape, Law No.5, 6.03.2000), GPS: 45°23′33.95″ N; 25°30′26.41″ E, elev. 800–960 m a.s.l., currently in Prahova and Dămbovița Counties, Southern Carpathians, Central Romania. This species requires further observation to determine whether other known populations are present in other areas within the Bucegi Mountains Natural Park protected area (Figure 1C).

**Population counts:** The population (2009–2011) contained ca. 5–6(7) individuals in Prahova County, Bucegi Natural Park; two additional populations (2022) contained a total of ca. 60–75 individuals in Dămbovița County, Bucegi Natural Park.

**Area of occupancy (AOO):** Greater distribution area ca. 45–50.5 km^2^ (micro-endemism); GPS: 45°23′33.95″ N; 25°30′26.41″ E, elev. 800–960 m a.s.l., currently covering parts of Prahova and Dămbovița Counties, Bucegi Natural Park (Figure 1C). 

**Examined material:** Romania. Bucegi Mountains Natural Park ROSCI001 protected area Natura 2000: forested, subalpine area of Șipotului Valley of lower Dămbovița County, Southern Carpathians, elev. 860–890 m, 17–21 July 2022, fl. ca. 12–26 July 2022, *NE Anghelescu NEA410357* (Holotype: BUC–barcode 401357) [currently at the Herbarium of the Botanical Garden Bucharest].

**Voucher:** The voucher specimen was deposited at the Herbarium of the Botanical Garden Bucharest (BUC!).

**Etymology:** The epithet *bucegensis* chosen for the new species is derived from the name Bucegi, ad litteram meaning of Bucegi, a reference to the Natural Park and the mountain range where the species was discovered.

**Proposed conservation status:** Endangered (EN). *Epipactis bucegensis* has only been reported from the Bucegi Natural Park ROSCI001 protected area Natura 2000, Prahova and Dămbovița County, Southern Carpathians, Romania. The two newly found populations, containing a total of ca. 75 individuals, were found in an area no greater than 45–50 km^2^. Nevertheless, we take into consideration that more future research in other subalpine areas of the park may lead to discovering new populations of *Epipactis bucegensis*. In recent years, Bucegi Natural Park proved to harbour undiscovered taxa, such as the newly discovered *Nigritella nigra* subsp. *bucegiana* Hedrén, Anghel. and R.Lorenz, subsp. nov. [87]. At the same time, our future research includes several similar habitats outside the Bucegi Mountains Natural Park protected area that may be suitable to *Epipactis bucegensis* occurrence, since they are important biological reserves for threatened species [88]. It must, however, be emphasized that this micro-endemism is restricted to an area subject to rapid deforestation due to abrupt urban expansion and increased anthropogenic activities, such as cattle farming, agriculture, tourism and real estate development. According to the EU Biodiversity Strategy (2020–2050), which works towards restoring natural environments by stopping the destruction of ecosystems and loss of biodiversity [89], effective measures should be implemented in order to protect and preserve these fragile habitats that harbour rare endemic species. Consequently, we are proposing this taxon, which is restricted exclusively to one mountain range, to be treated as ‘Endangered’ (EN) following the Red List criteria of the IUCN Standards and Petitions Committee of the IUCN [90].

## Figures and Tables

**Table 1:** Morphologic differences and morphological comparisons of the novel, obligately autogamous taxon, *Epipactis bucegensis*, *Epipactis muelleri* Godfery and its relative, the allogamous *Epipactis helleborine* (L.) Crantz.

Vegetative and Floral Organs	Characters/Features	*Epipactis bucegensis*		
	(Millimetres)		*Epipactis muelleri*	*Epipactis helleborine*
1. Rhizome				
	Rhizome length	40–120 (160)	NA	50–150 (180)
	Rhizome diam.	13–25 (31)	NA	13–26 (38)
2. Adventitious Roots				
	Adv. roots length	20 (34)–100 (124)	NA	20 (34)–100 (124)
	Adv. roots diam.	1.5–2.0 (3.2)	NA	1.5–2.0 (3.2)
3. Stem				
	* Stem type	* Spindly, flexuous, yellowish-green	Spindly, greenish	Strong, robust, green
	Overall height	180–300 (500)	(10) 200–350 (400)	(200) 350–900 (1300)
	Stem diameter	3.0–5.0 (8.0)	3.0–4.3 (6.9)	5.0–9.0 (14)
	Stem anthocyanins	Absent	Absent	Present basally
	* Trichomes (Glandular hairs)	* Densely glandular–pubescent along the entire stem	Basal glandular–pubescent	Basal glabrous, downy towards the tip
4. Leaves				
	* Basal sheath	* 1, acuminate–lanceolate, tapering at tip, yellowish-green	1, acuminate–elongate, light green	1, round–ovoidal, wide around the middle, green
	Basal sheath length	15–25	14 (16)–22 (25)	18–31
	Basal sheath width	9.0–20	7.0 (9)–15 (20)	10–25
	* Distribution of sheathing leaves on stem	* Numerous in the lower half	Numerous in the middle part	Numerous in the middle part of the stem
	Phyllotaxy	Alternate	Alternate	Alternate
	* Longest leaf posture	* Spreading to erect, to a subtended angle of c. 30° relative to the stem (angled)	Spreading horizontally to curved downwards, arched	Spreading horizontally to a subtended angle of c. 90° relative to the stem (perpendicular to the stem)
	* No. of basal cauline leaves	* 3–4 (8), elongate, acuminate	1 (3), ovoid–elongate, wide, arched, acuminate	1 basal sheath (see above)
	No. of median cauline leaves	3 (5), elongate–oval, acuminate	5 (8), ovoid–elongate, wide, arched, acuminate	4 (10), ovoid–orbicular
	No. of upper cauline leaves	1–2 (4), narrow–lanceolate acuminate	1–2, narrow–lanceolate acuminate	3 (4), lanceolate acuminate
	Length of longest leaf	90 (120)	33–100 (125)	100 (140)
	Width of longest leaf	35 (40)	1.9–3 (4.3)	60 (75)
	Outline shape of longest leaf	Oval to elongate–lanceolate, broadest in the middle	Ovoid–elongate, acuminate, broadest in the middle	Orbicular to ovoid–elongated, broadest in the middle
	* Leaf conduplicate	* Strongly keeled	Keeled	Moderately keeled
	* Apical hooding	* Moderate	Absent	Absent
	* Leaf colour	* Yellowish-green to green (aged plants)	Light- to deep-green	Green to dark-green
	* Leaf dorsal side	* Yellowish	Light- to deep-green	Green to dark-green
	* Leaf ventral side	* Shiny, yellowish-green	Shiny, light-green	Shiny, deep-green
	Leaf margins	Undulate, edged with fine papillae	Undulate	Entire, straight, edged with fine papillae
	Leaf markings	Unspotted	Unspotted	Unspotted
	* Leaf venation	* Strongly veined	Veined	Veined
	Upmost leaf	Bract-like, narrow–acuminated, yellowish	Bract-like, narrow–acuminated, greenish	Bract-like, deep- to dark-green
5. Bracts				
	Length of basal bracts	15–38 (45) exceeding the flowers	12–33 (45) exceeding the flowers	35–58 (102), exceeding flowers
	Width of basal bracts	3.2 (4.2)–8.0 (12)	3.2–5.4 (8.3)	19–(24) 32
	Length of floral bracts	18–22 longer than the ovaries, shorter than the flowers	19–25 longer than the ovaries, shorter than the flowers	28–41 exceeding flowers
	Width of floral bracts	2.0 (3.0)–4.0 (8.0)	2.0 (2.8)–3.6 (6.5)	12 (23)–14 (28)
	Texture of bracts	Robust, keeled	Membranous	Robust to membranous
	Bract anthocyanins	Absent, bracts yellowish	Absent, bracts light green	Absent, bracts deep-green
	Bract margins	Entire, undulation missing, edged with tooth-like papillae	Entire, undulation missing, edged	Entire, undulation missing, edged with tooth-like papillae
6. Inflorescence	Inflorescence length	100–150	100 (200)–250 (300)	100–200 (450)
	Inflorescence diameter (calculated from bract-tip to bract-tip)	35–45 (55)	25–35 (45)	35–55 (95)
	Inflorescence shape	Elongated raceme, near-one-sided, lax to dense	Elongated raceme, lax, near-one-sided	Elongated raceme, near-one-sided, dense
	No. of flowers	10–30 (50/65)	4 (10)–15 (35)	10–30 (70/100)
7. Flowers	Flower type	Cleistogamous, half- to completely closed, pendant	Cleistogamous, half-opened, pendant	Chasmogamous, wide-opened, near-erect
	Flower length	8.5–11.2	7.2–9.0	8.8–14.3
	Flower diameter	4.5–6.5	3.3–5.5	4.8–7.8
	Flower colour	Whitish-yellow to yellowish-green (aged individuals)	Yellowish-green	Greenish, purple, violet, pink, brownish
8. Scent		Absent	Absent	Faint to moderate, sweet to fermented
9. Sepals				
	Sepal shape	Ovoid–elongated	Ovoid–elongated	Ovoid–roundish
	Sepal no. and colour	3, Whitish-yellowish	3, Yellowish-green	3, Greenish, violet-purple tinged
	Sepal apex	Acuminate, tapering	Acuminate, tapering	Roundish–acuminate
	Sepal length	7.0–14.2	5.0 (6.5)–10.2 (11)	7.2–14.2
	Sepal width	5.5–7.1	3.9–4.5	4.5–7.5 (8.8)
10. Lateral petals	Petal no. and colour	2, Whitish-yellowish	3, Yellowish-green to pinkish-green	2, Greenish, violet-purple, brownish-purple tinged
	Lateral petal shape	Oval–lanceolate, tapering	Oval–lanceolate	Roundish–oval lanceolate
	Lateral petal apex	Acuminate elongated, tapering	Acuminate elongated, tapering	Acuminate elongated, tapering
	Lateral petal length	6.0–9.0	4.9–6.2 (7.1)	6.9–12.5 (13.5)
	Lateral petal width	4.0–6.1	3.0–4.1 (5.50)	4.0–6.1 (6.9)
11. * Labellum				
	Outline shape	Orbicular, wide, flat, bipartite	Orbicular, tripartite	Orbicular, tripartite
	* Hypochile shape	* Wide, ovoid	Orbicular, cup-like	Orbicular, cup-like
	Hypochile length	3.0–4.1	3.2–3.8 (4.2)	2.1 (3.0)–3.9
	Hypochile width	4.0–5.2	3.6–4.5 (5.1)	3.8–4.2
	* Hypochile inner wall colour	* Shiny, crimson-purple to brownish-purple	Shiny, blackish-brown	Shiny, blackish-brown to blackish-green
	* Mesochile	* Completely absent	Well-defined, wide junction	Well-defined, narrow junction
	* Epichile shape	* Triangular, flat, tapering	Obtuse, heart-shaped, roundish–wide at the apex	Heart-shaped, obtuse, turned downwards
	Epichile length	5.0–5.8	4.5–5.5 (6.1)	5.8.0–6.7 (7.1)
	Epichile width	3.0–4.5	3.9–4.0	3.1–4.2 (5.9)
	* Epichile colour	* White	Yellowish-green, purple tinted	Greenish-purple, brownish
	* Epichile lateral lobes	* Absent to mildly defined	Defined, ovoid-roundish, scalloped	Defined, ovoid–roundish
	* Median lobe	* Triangular, flat, tapering	Triangular, roundish, obtuse	Roundish, obtuse, downcurved
12. * Calli	* Basal calli no. and colour	* 2, pyramidal, tooth-like, prominent, wide apart, crimson-purple, non-wrinkled	2, less-prominent, wrinkled, attenuated, greenish-yellow to pinkish, closely placed	2, prominent, wrinkled, well-developed, variously coloured
	Central groove	Present, well defined with a minute basal callus	Present, greenish	Present, well-defined, darker
	* Central lobe apex	* Narrow, acuminate/tapering, elongated	Roundish, wide, obtuse	Roundish, wide, obtuse
	* Base colour of median lobe	* White	Greenish-yellow	Variously coloured—green, violet, brownish
	* Margin colour	* White	Greenish-yellow	Variously coloured
	Lateral lobes	Scalloped, poorly defined to completely absent	Scalloped, moderate indentation	Entire/moderate indentation, scalloped, slightly recurved
	Lateral lobe margins	White, entire, scalloped	Greenish, entire, scalloped	White, entire to moderate indentation
13. Spur				
	Presence	Absent	Absent	Absent
14. Nectar				
	Presence	Absent-to-minute droplets in topmost flowers	Present in fair amounts	Present in abundant amounts
15. Gynostemium				
	Type	Thick, cylindrical	Thick	Thick
	Colour	White, shiny	Creamy-white	Creamy-white
16. Staminodes				
	Presence	Present, wing-like	Well-defined, wing-like, large	Well-defined, wing-like, large
17. Auricles				
	Presence	Absent	Absent	Absent
18. Clinandrium				
	Presence	Absent	Absent	Present, well-defined
19. Anther				
	Anther colour	Translucent white	Creamy-white	Creamy-white to white
	Anther shape	Broad, short	Broad, short	Broad, short
	Type	Sessile, broad, bithecal	Sessile, broad, bithecal	Stalked, bithecal
	Angle relative to stigma	Angled, pushed forward	Angled, pushed forward	Erect
20. * Anther cap				
	* Anther cap shape	* Elongated, fleshy, tapering	Elongated, fleshy, acuminate	Elongated, fleshy, tapering to roundish
	* Anther cap colour	* Whitish-creamy	Yellowish	
	Type	Well-developed, bithecal	Well-developed, bithecal	Well-developed, bithecal
21. Pollinia				
	Shape	Elongated, ovoid, bipartite	Elongated, ovoid, bipartite	Elongated, clavate, bipartite
	Pollinia length	1.5–2.3	1.6–2.5	1.8–2.9 (3.1)
	Pollinia width	0.5–1.2	0.6–1.4	0.5–1.4 (1.8)
	Compactness	Mealy, friable, disintegrating	Moderately compact to friable	Compact, non-disintegrating
	Contact with stigma	Stigmatic contact before anthesis	Stigmatic contact before anthesis	No stigmatic contact
22. Viscidium				
	Presence	Absent	Absent	Present, well-developed, sac-like, milky
23. * Stigma				
	* Position relative to the gynostemium	* Perpendicular, roof-like, entirely flat	Perpendicular, deeply V-shaped, deeply concave	Parallel to near-parallel, slightly concave
	*Shape	* Rectangular, wider than longer, bilobed (rostellum absent), roof-like, entirely flat	Quadrangular, bilobed (rostellum absent), deeply V-shaped, deeply concave	Quadrangular, trilobed
	Stigmatic exudate	Highly abundant	Abundant	Fair amounts
24. Rostellum				
	Presence	Absent	Absent	Present, well-developed (median stigmatic lobe) forming the upper stigmatic rim
25. Bursicles				
	Presence	Absent	Absent	Absent
26. Ovary				
	Ovary shape and colour	Pear-shaped, round apex, strongly ridged	Elongated, spindle-shaped, light-green, non-ridged	Pear-shaped, mildly ridged
	Ovary colour	Dark green, mildly purple pigmented (anthocyanins)	Light-green	Green to dark green
	Ovary length	6.2–8.5	6.2 (7.3)–8.8 (9.0)	5.2–9.5
	Ovary diameter	3.9–4.8	3.2–4.0 (4.5)	3.7–5.9
	* Ovary colour	* Yellowish-green to deep-green in fruiting plants	Green	Green to dark-green
27. Flower pedicel				
	Pedicel length	1.2–2.5		1.6–2.9
	Pedicel diam.	1.0–1.2		1.0–1.4
	* Pedicel pigmentation	* Faint violet-washed at base (entirely greenish)	entirely greenish, with no basal pigmentation	Purple-pigmented at base
	* Pedicel colour	* Yellowish-green	Greenish	Greenish-violet basally
	Pedicel pubescence	Downy, covered in glandular hairs (trichomes)	Absent to mildly pubescent	Downy, covered in glandular hairs (trichomes)
28. * Fruit				
	* Fruit shape	Club/*ear-shaped, swollen, ovoid with round apex, strongly ridged	Pear-shaped, elongated-ovoid, smooth	Pear-shaped, swollen middle part, mildly ridged
	* Fruit colour	Green to dark green (aged plants), purple washed at the base	Light-green	Green to dark green (aged plants)
	Fruit length	6.9–10.5 (12.3)	6.9–10.5 (12.3)	5.9–10.4 (15.3)
	Fruit width	4.9–5.9 (6.3)	4.9–5.9 (6.3)	4.7–6.1 (6.8)
	Fruiting	July–August	June–July	July–August
	Fruit set	70–95%	10–30% (60%)	60–78%
29. Seed capsule				
	Capsule length	7.1–12 (13.1)	6.8–9 (10.5)	6.2–12.5 (14.1)
	Capsule width	5.1–6.5	4.8–5 (6.2)	5.5–7.2
	Capsule colour	Brownish	Brownish	Brownish
	Capsule maturation	September–October	September–October	September–October
30. Flowering time		July	June	July–August
31. Pollination strategy				
	Pollination type	Non-entomophilous	Non-entomophilous	Entomophilous
	Pollination mode	Self-pollinating	Self-pollinating	Cross-pollinating
32. Reproductive strategy				
	* Reproductive strategy type	* Obligate autogamous	Obligate/Facultative autogamous	Obligate allogamous

* (asterisk) marks the features that significantly differentiate *Epipactis bucegensis* from the related *Epipactis* species.

## Data Availability

No data availability.

## Abbreviations

**a.s.l.—**above sea level; **ac**—anther cap; **anc**—anther connective; **AOO**—area of occupancy; **ath**—anther theca; **BCE**—before current era; **BNP**—Bucegi Natural Park; **br**—bract; **ca**—callus; **CDP**—composite dissection plate; **cli**—clinandrium; **dhl**—dehiscence lines; **elev.**—elevation; **EOO**—extent of occurrence; **epc**—epichile; **fr**—fruit; **gpt**—germinating pollen tetrads; **gyn**—gynostemium; **hyc**—hypochile; **ipt**—individual pollen tetrads; **lsl**—lateral stigmatic lobe; **msc**—mesochile; **ne**—nectar; **ov**—ovary; **pe**—petiole; **po**—pollinia; **pol**—pollinarium; **ros**—rostellum; **s.l.**—sensu lato; **s.s.**—sensu stricto; **st**—stigma; **sta**—staminodium; **tfl**—thecal flaps; **trh**—trichomes; **vis**—viscidium.
